# Bridging the Structural Gap: A Methodological Review of Cryo-Electron Microscopy for Underrepresented Viruses

**DOI:** 10.3390/v18020195

**Published:** 2026-02-01

**Authors:** Yoon Ho Park, Hyun Suk Jung, Sungjin Moon, Chihong Song

**Affiliations:** 1 Department of Biochemistry, College of Natural Sciences, Kangwon National University, Chuncheon 24341, Republic of Korea; yhpark99@kangwon.ac.kr (Y.H.P.);; 2 Department of Biological Science, Kangwon National University, Chuncheon 24341, Republic of Korea; 3 Department of Convergence Medicine, School of Medicine, Pusan National University, Yangsan 50612, Republic of Korea

**Keywords:** Cryo-EM, vertebrate viruses, insect viruses, plant viruses, giant viruses

## Abstract

Cryo-electron microscopy (cryo-EM) has revolutionized structural virology, enabling routine structure determination at 2–4 Å resolution, with exceptional cases reaching 1.56 Å. The structural diversity of viruses across vertebrate, plant, and insect hosts provides fundamental insights into infection mechanisms, host–pathogen coevolution, and therapeutic target identification. However, analysis of Electron Microscopy Data Bank entries reveals notable disparities in structural coverage: among 11,717 eukaryotic virus structures (excluding bacteriophages), vertebrate viruses constitute 97.6% (*n* = 11,432) of deposited entries, while plant viruses (1.0%; n = 117) and insect viruses (1.4%; n = 168) remain significantly underrepresented. This bias stems from distinct technical barriers including size limitations for giant viruses exceeding 200 nm, the loss of asymmetric information during symmetry-imposed processing, and the morphological complexity of filamentous and pleomorphic viruses. Each barrier has driven the development of specialized methodological solutions: block-based local refinement overcomes through-focus variations in giant viruses, cryo-electron tomography (cryo-ET) validates and reveals asymmetric features lost in symmetrized reconstructions, and subtomogram averaging enables structural analysis of pleomorphic assemblies. This review synthesizes recent methodological advances, critically evaluates their capacity to address specific technical barriers, and proposes strategies for expanding structural investigations across underrepresented host systems to achieve comprehensive understanding of viral structural biology.

## 1. Introduction to Host-Specific Barriers in Viral Structure

Viruses represent one of the most diverse biological entities, infecting virtually every form of cellular life across vertebrate, plant, and insect kingdoms [1]. Understanding the structural basis of viral infection requires atomic-level resolution of viral components, from capsid proteins to envelope glycoproteins. Structural virology has undergone a transformative revolution in cryogenic electron microscopy (cryo-EM), evolving from a technique once termed “blobology” to a method capable of achieving near-atomic to atomic resolution structures [2,3].

The importance of characterizing virus structures across diverse host systems cannot be overstated. Animal viruses, particularly those causing human disease, have received disproportionate attention due to their direct medical relevance and associated research funding. Plant viruses, despite causing estimated annual crop losses exceeding $30 billion globally [4], remain comparatively understudied at the structural level. Insect viruses, including those infecting model organisms such as *Drosophila melanogaster*, offer unique opportunities to understand fundamental aspects of viral evolution and innate immunity, yet face substantial barriers limiting their structural characterization.

This review critically examines the current landscape of host-specific virus structure determination, analyzing the technical barriers that have created disparities in structural coverage across host systems. By identifying the specific challenges posed by viral size, symmetry, and morphology, and evaluating the methodological solutions developed to address each barrier, we aim to provide a framework for achieving more comprehensive structural coverage of the virosphere.

## 2. Host-Specific Bias in Structural Databases

**Figure 1:**
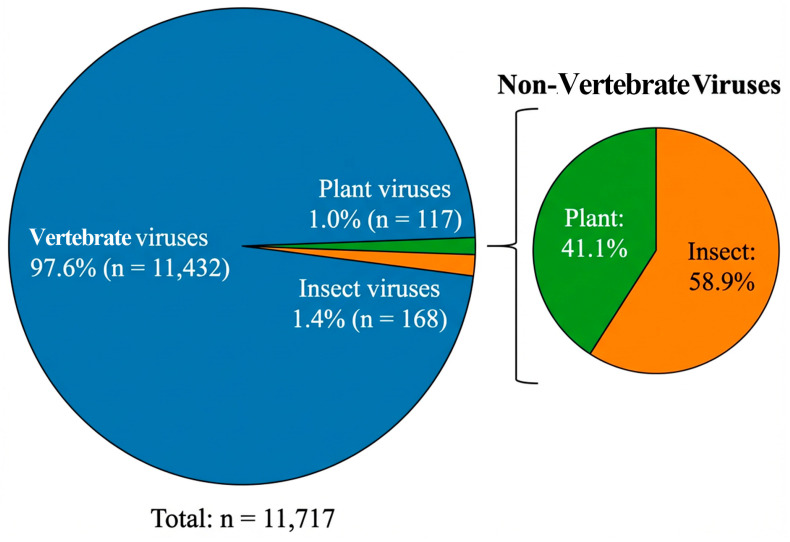
Host-specific distribution of eukaryotic virus structures in the Electron Microscopy Data Bank (EMDB). Host-specific distribution of eukaryotic virus structures (n = 11,717) in the Electron Microscopy Data Bank (EMDB). Analysis of EMDB entries (accessed December 2025) reveals notable disparities in structural coverage across virus host categories.

Analysis of Electron Microscopy Data Bank (EMDB) entries (accessed December 2025) reveals notable disparities in structural coverage across virus host categories (Figure 1). Of 12,875 total virus-related entries, 1158 correspond to bacteriophages, leaving 11,717 eukaryotic virus structures. Among these, vertebrate viruses dominate at 97.6% (n = 11,432), while plant viruses account for merely 1.0% (n = 117) and insect viruses 1.4% (n = 168) (Figure 1). This reflects a complex interplay of funding priorities, research infrastructure, and intrinsic technical challenges associated with each virus category.

### 2.1. Vertebrate Viruses

Vertebrate viruses, particularly those causing human disease, represent the most extensively characterized category in structural virology. This dominance reflects decades of sustained research investment driven by public health priorities and the infrastructure of medical research institutions. The extensive characterization of medically relevant families including Coronaviridae, Flaviviridae, Orthomyxoviridae, and Herpesviridae has established a rich structural foundation for understanding viral pathogenesis [5,6,7].

Vertebrate viruses exhibit structural diversity, ranging from small icosahedral particles (parvoviruses, ~25 nm) to large enveloped virions (herpesviruses, ~200 nm) and pleomorphic assemblies (orthomyxoviruses, influenza) [8]. Many medically important vertebrate viruses possess well-ordered icosahedral symmetry amenable to single-particle analysis, enabling routine high-resolution structure determination [9]. However, significant subsets including retroviruses (HIV), paramyxoviruses, and filoviruses exhibit pleomorphic enveloped morphologies that require specialized tomographic approaches [10].

Recent achievements demonstrate the capabilities of modern cryo-EM for vertebrate virus characterization. The determination of adeno-associated virus at 1.56 Å resolution represents a landmark where individual hydrogen atoms and ordered water molecules become visible [11,12]. Beyond static structures, cryo-EM has captured functional conformational dynamics, including the “rising” and “resting” conformations of norovirus P-domains and portal-like assemblies formed by calicivirus VP2 following receptor engagement [13,14].

### 2.2. Plant Viruses

Despite their substantial agricultural and economic impact, plant viruses constitute only 1.0% of EMDB eukaryotic virus structure entries, a mere 117 structures compared to over 11,000 vertebrate virus structures. This underrepresentation persists despite plant viruses causing estimated annual global crop losses exceeding $30 billion and threatening food security in developing regions. The reflects systematic biases in research funding that prioritize human and veterinary pathogens, as well as distinct technical challenges specific to plant virus structural biology [15].

The structural landscape of plant viruses has historically been dominated by well-behaved model systems. Tobacco mosaic virus (TMV), bromoviruses, tombusviruses, and geminiviruses have received moderate structural attention due to their amenability to high-yield purification and their rigid icosahedral or well-ordered helical symmetries [16,17,18,19]. However, these models do not represent the majority of agriculturally significant pathogens, particularly the flexuous filamentous viruses that cause the bulk of viral crop diseases.

Plant viruses display distinctive morphological features that distinguish them from most vertebrate viruses. A disproportionate number of economically important plant viruses, particularly members of Potyviridae (the largest family of plant-infecting RNA viruses), Closteroviridae, and Flexiviridae, exhibit flexuous filamentous morphologies rather than compact icosahedral shapes [20]. These elongated, flexible particles present significant challenges for high-resolution structure determination, particularly in image processing, due to their inherent conformational heterogeneity [21]. Furthermore, plant viruses encode movement proteins to facilitate cell-to-cell transport through plasmodesmata. Although these proteins represent unique structural targets not found among vertebrate viruses, their tight association with cellular membranes creates substantial hurdles for isolation and structural characterization [22].

Notably, the structure of sweet potato feathery mottle virus (Potyviridae) was determined at 2.9 Å resolution, revealing the conserved coat protein fold and its interaction with the single-stranded RNA genome [23]. Similarly, structures of sweet potato mild mottle virus and Pepino mosaic virus have been resolved at 2.6 Å and 3.9 Å, respectively, providing important insights into the assembly mechanisms of flexuous virions [23,24]. These breakthroughs demonstrate that the technical barriers limiting plant virus structural biology are becoming surmountable.

### 2.3. Insect Viruses

Insect viruses represent another underexplored category in structural virology, constituting only 1.4% (n = 168) of EMDB eukaryotic virus structure entries despite insects comprising the most species-rich animal class. Although insect viruses offer unique biological insights and hold practical value for biological pest control and recombinant protein expression systems, they remain significantly underrepresented in structural databases [25].

Insect viruses encompass structural diversity, including large occluded baculoviruses with complex biphasic life cycles, small RNA viruses (Dicistroviridae, Iflaviridae), and unique double-stranded RNA viruses (Birnaviridae, Reoviridae) [26,27]. Many insect-specific virus families lack close relatives among vertebrate viruses, potentially harboring novel protein architectures not represented in current structural databases. Baculoviruses are particularly notable for their occlusion bodies crystalline protein matrices embedding rod-shaped nucleocapsids representing a structural form unique among viruses [28].

In the context of host–pathogen interactions, *Drosophila melanogaster* serves as a powerful genetic model for dissecting innate immunity, particularly RNA interference pathways and virus–host coevolution [29]. The fruit fly lacks adaptive immunity, making it an ideal system for understanding fundamental antiviral mechanisms conserved across arthropods [30]. However, despite the extensive genetic characterization of the host, the structural biology of its native viruses remains relatively limited. While the structure of *Drosophila C virus* (DCV) has been well-characterized, providing insights into capsid stability and genome release, many other *Drosophila*-specific viruses identified through metagenomics remain structurally undefined [31]. Expanding structural studies to these neglected viruses is essential to fully exploit *Drosophila* as a comprehensive model for viral pathogenesis.

## 3. Technical Barriers Driving the Bias

The disparities in structural coverage across host systems arise not only from funding and priority differences but also from distinct technical barriers that differentially affect plant and insect viruses (Table 1). Understanding these barriers is essential for developing targeted solutions and achieving comprehensive structural coverage of the virosphere.

### 3.1. Size Barriers: Ewald Sphere Curvature and Depth of Field Effects in Giant Viruses

Giant viruses, including members of the Nucleocytoplasmic Large DNA Virus (NCLDV) superfamily, present fundamental physical challenges that standard cryo-EM approaches cannot overcome [32]. As viral dimensions increase beyond 200 nm, two related physical phenomena become limiting factors for high-resolution structure determination.

The electron microscope focuses on a specific plane within the sample. For giant viruses, the top and bottom of the capsid are significantly defocused relative to the central plane, introducing blur that degrades high-resolution information. This “depth of field” problem worsens proportionally with particle size, creating an effective resolution ceiling for large assemblies imaged using standard protocols [33].

In electron scattering, the assumption that the Ewald sphere can be approximated as a plane becomes invalid for large objects at high resolution. The curvature of the Ewald sphere introduces phase errors that manifest as resolution anisotropy and blurring in reconstructions [34]. For giant viruses, these effects become non-negligible at resolutions better than approximately 5–7 Å, historically limiting structures to moderate resolution insufficient for atomic model building.

While giant viruses are found across all host categories, certain plant-infecting NCLDVs (phycodnaviruses infecting algae) and insect-infecting viruses (iridoviruses, entomopoxviruses) fall into the giant virus category. The size barrier has historically prevented high-resolution structural analysis of these important virus families, contributing to the underrepresentation of plant and insect viruses in structural databases.

### 3.2. Symmetry Barriers: Obscuring of Functional Asymmetry by Icosahedral Averaging

Single-particle analysis (SPA) of icosahedral viruses routinely exploits 60-fold symmetry during reconstruction to significantly improve signal-to-noise ratios [35]. While this symmetry imposition enables high-resolution structure determination from modest dataset sizes, it creates a fundamental barrier: biologically relevant asymmetric features are averaged away during processing, resulting in loss of asymmetric information that may be functionally critical [36].

Recent studies have revealed that even viruses assumed to possess perfect icosahedral symmetry exhibit imperfect icosahedral symmetry when analyzed without symmetry imposition [37]. Alphaviruses, previously characterized exclusively by symmetry-imposed single-particle analysis, show asymmetric features that are functionally relevant but completely absent in symmetrized reconstructions. These include genome organization patterns, unique vertices associated with portal complexes, and localized structural heterogeneity.

Many plant viruses package their genomes in specific orientations or utilize unique vertices for genome delivery features invisible under imposed symmetry [38]. Insect viruses, particularly those with complex life cycles involving different host cell types, may exhibit structural variations between morphological forms that symmetry averaging obscures. The reliance on symmetry-imposed processing has potentially masked important biological insights for these underrepresented virus categories.

### 3.3. Morphology Barriers: Structural Heterogeneity and Flexibility in Filamentous and Pleomorphic Viruses

Standard single-particle analysis is optimized for compact, roughly spherical particles that can be accurately aligned and averaged. Viruses with non-spherical morphologies particularly filamentous and pleomorphic forms present distinct challenges that have historically limited their structural characterization.

Flexuous filamentous viruses, which include many of the most economically important plant pathogens (Potyviridae, Closteroviridae, Flexiviridae), exhibit continuous bending and twisting along their length. This conformational heterogeneity prevents straightforward helical reconstruction, as different segments of the same particle adopt different conformations. Additionally, filament length heterogeneity and end effects complicate particle selection and averaging strategies. Standard helical reconstruction assumes rigid, straight particles an assumption violated by flexuous viruses [39].

Enveloped viruses such as influenza, HIV, and baculoviruses incorporate host-derived lipid membranes and display surface glycoproteins in irregular patterns. These pleomorphic viruses vary in size, shape, and surface protein arrangement between individual particles, precluding the averaging strategies that enable high-resolution single-particle analysis. Each particle is essentially unique, eliminating the fundamental assumption of particle homogeneity underlying SPA.

The morphology barrier disproportionately affects plant viruses, where flexuous filamentous morphology predominates among economically important pathogens. For insect viruses, in the budded form of baculoviruses, the morphological form mediating systemic infection is pleomorphic and enveloped, explaining why baculovirus structural studies have focused predominantly on the occluded form despite the biological importance of budded virions. These morphological barriers have been primary drivers of the underrepresentation of plant and insect viruses in structural databases.

## 4. Methodological Solutions

Each technical barrier has driven the development of specialized methodological solutions. Understanding which method addresses which barrier enables strategic application of these approaches to underrepresented virus systems.

### 4.1. Block-Based Reconstruction: Mitigating Ewald Sphere and Focus Gradients via Local Refinement

The block-based reconstruction approach (also termed local refinement or divide-and-conquer strategy) computationally divides large viral particles into smaller blocks, each treated as an independent particle for local defocus refinement and reconstruction [34,40]. Within each small block, the assumptions of planar CTF and negligible Ewald sphere curvature hold true. The locally refined blocks are subsequently reassembled to generate the complete viral structure, effectively bypassing the physical limitations that arise at the whole-particle level.

Application of block-based reconstruction has enabled near-atomic resolution structures of multiple giant viruses. Singapore grouper iridovirus (SGIV), with a diameter of approximately 228 nm, was resolved at 3.5 Å, revealing the architecture of its major and minor capsid proteins [41]. Medusavirus capsid maturation was characterized at 7.3–9.9 Å resolution, providing insights into pentascale structural transitions [42]. Jyvaskylavirus, the first giant virus isolated from Finland, further demonstrates expanding structural coverage of NCLDVs [43]. The method has also been applied to jumbo bacteriophages, resolving the capsid structure of bacteriophage ΦKZ [44].

Block-based reconstruction introduces specific trade-offs. Computational costs scale with particle size, as each block requires independent processing. Block boundary effects can potentially introduce discontinuities at interfaces between locally refined regions, requiring sufficient overlap between blocks and careful validation. The method assumes blocks are sufficiently small to approximate planar CTF behavior, an assumption requiring verification for the largest viruses or highest target resolutions.

Block-based reconstruction directly enables structural analysis of giant plant-infecting viruses (phycodnaviruses) and giant insect-infecting viruses (iridoviruses, entomopoxviruses) that were previously intractable. Systematic application to these underrepresented giant virus families could increase structural coverage.

### 4.2. Cryo-Electron Tomography: Recovering Asymmetric Information via Symmetry-Free Reconstruction

Cryo-electron tomography (cryo-ET) collects a tilt series of images of the same specimen area at different angles, enabling reconstruction of three-dimensional tomograms without imposing any symmetry [45]. Each viral particle is reconstructed individually, preserving asymmetric features that would be averaged away in single-particle analysis. By comparing symmetry-imposed SPA structures with cryo-ET reconstructions, researchers can identify structural features lost during symmetrized processing.

Studies employing cryo-ET validation have demonstrated that imperfect icosahedral symmetry is more common than previously appreciated. In alphaviruses, cryo-ET revealed asymmetric features that are functionally relevant but completely absent in symmetrized reconstructions [46]. Flaviviruses similarly exhibit asymmetric positioning of the nucleocapsid core relative to the glycoprotein shell [47]. Similar observations have been made for genome organization within viral capsids, where asymmetric nucleic acid packaging cannot be visualized under imposed symmetry [38]. These findings emphasize the importance of cryo-ET as a complementary validation technique.

Historically, cryo-ET achieved lower resolution than SPA for whole virions, but recent advances in STA have reached 3–4 Å resolution for symmetrized subunits, approaching the 2–4 Å routinely achieved by SPA [45,48,49]. Tilt-series collection increases radiation damage and data acquisition time. The “missing wedge” of data due to limited tilt range enhances misalignment and contributes to potential anisotropic resolution. Despite these limitations, cryo-ET provides essential validation of symmetry-imposed structures.

Cryo-ET validation is particularly valuable for plant viruses, where genome packaging orientation and portal complexes may be functionally critical yet invisible under imposed symmetry. For insect viruses with complex life cycles, asymmetric features distinguishing different morphological forms can now be characterized.

### 4.3. Subtomogram Averaging: Resolving Structural Heterogeneity Through Local Unit Alignment

Subtomogram averaging (STA) combines the three-dimensional imaging capability of cryo-ET with averaging of repeating structural units. After tomogram reconstruction, repetitive elements such as envelope glycoprotein spikes, capsid subunits, or helical segments are computationally extracted as “subtomograms” and aligned for averaging [50]. This approach does not require global particle homogeneity; instead, local structural units are averaged while preserving information about their spatial arrangement within heterogeneous assemblies.

STA has enabled structural analysis of previously intractable pleomorphic viruses. In situ visualization of influenza A virus assembly captured the spatial arrangement of hemagglutinin (HA) and neuraminidase (NA) on budding virions directly within host cellular context [51]. Herpesvirus capsid assembly intermediates were resolved within the cell nucleus, redefining canonical assembly pathways [52,53]. The membrane replication factories of hepatitis C virus (HCV) and Zika virus have been characterized, revealing potential targets for antiviral intervention [54,55].

For flexuous filamentous viruses, STA offers an alternative to conventional helical reconstruction. By extracting and averaging short segments rather than assuming global helical order, STA can accommodate the bending and conformational heterogeneity characteristic of plant flexiviruses. Recent applications to filamentous plant viruses have achieved sub-nanometer resolution while accommodating the flexibility that precluded earlier structural analysis [23].

Resolution achieved by STA has historically been 5–15 Å, with recent advances reaching 3–4 Å for symmetrized viral subunits [48,49,50,56]. Despite these improvements, computational demands for tomogram reconstruction and subtomogram alignment remain substantial, though hardware acceleration and improved algorithms continue to reduce these barriers [50]. Computational demands for tomogram reconstruction and subtomogram alignment remain substantial, though hardware acceleration and improved algorithms continue to reduce these barriers. The requirement for tilt-series collection limits throughput compared to single-particle data acquisition.

STA directly addresses the morphology barrier that has significantly impacted plant virus structural coverage. The flexuous filamentous morphology characteristic of Potyviridae, Closteroviridae, and Flexiviridae families causing the majority of agricultural viral disease becomes tractable through STA approaches. For insect viruses, STA enables structural analysis of the pleomorphic budded form of baculoviruses, complementing existing knowledge of the occluded form.

**Figure 2:**
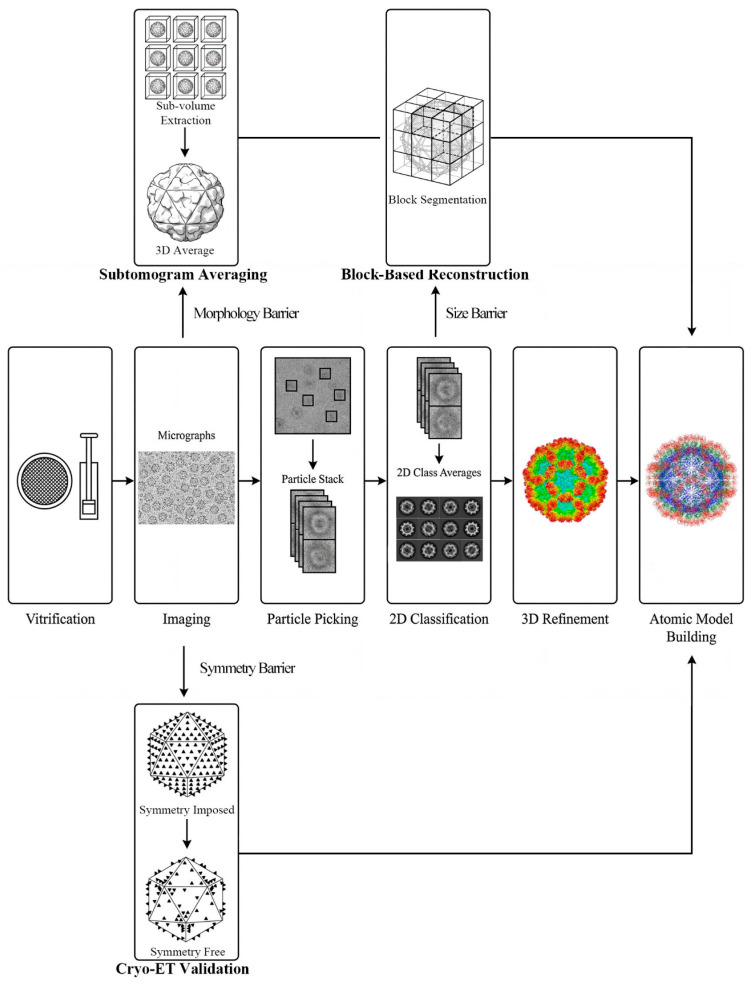
Integrated strategic workflow for the structural determination of underrepresented viruses. The central pathway depicts standard single-particle analysis (SPA) for typical icosahedral viruses, with schematic representations based on Sapovirus structure [57]. Specialized pathways overcome specific barriers: Subtomogram Averaging (STA) for morphological heterogeneity, Block-Based Reconstruction for size limitations, and Cryo-ET for symmetry validation. All strategies converge to a high-resolution atomic model.

Based on the technical characteristics discussed above, we propose a strategic decision-making workflow to guide the structural determination of underrepresented viruses (Figure 2).

## 5. Future Perspectives

The integration of in situ structural biology approaches represents the next frontier in viral structure determination. By combining cryo-focused ion beam (cryo-FIB) milling with cryo-ET, researchers can visualize viral infection processes within native cellular contexts [58]. This approach captures transient intermediates, host factor interactions, and membrane remodeling events invisible to purified particle analysis. For plant viruses, advances in cryo-FIB milling of plant tissues will enable visualization of plasmodesmatal transport and replication complex organization. For insect viruses, in situ approaches can reveal the cellular context of baculovirus assembly and the architecture of viral factories.

Recent demonstrations of high-resolution structure determination by cryo-EM at 100 keV accelerating voltage, together with high-resolution imaging on common 120 keV LaB6-source microscopes equipped with modern direct electron detectors, are lowering barriers to entry for the technique [59,60,61]. These developments will accelerate discovery of viral structures from diverse environmental niches and underrepresented host systems.

Integration of artificial intelligence (AI) and machine learning into cryo-EM workflows will revolutionize data processing. Automated particle picking, classification of heterogeneous assemblies, and structure prediction algorithms will accelerate structure determination while reducing operator bias [62,63]. For underrepresented virus families with limited prior structural information, AI-assisted approaches may prove particularly valuable by enabling structure determination from smaller datasets and facilitating model building for novel protein folds.

Emerging time-resolved cryo-EM approaches utilizing rapid mixing or light activation followed by rapid freezing promise to capture viral structural dynamics on millisecond timescales [64]. Visualization of receptor-triggered conformational changes, membrane fusion intermediates, and assembly pathways will provide mechanistic insights inaccessible to static structural snapshots [65].

## 6. Conclusions

This review has examined the host-specific bias in viral structural databases and the technical barriers driving this imbalance. The striking disparity in EMDB coverage vertebrate viruses (97.6%), plant viruses (1.0%), and insect viruses (1.4%) reflects not merely funding priorities but fundamental technical challenges that differentially affect virus categories.

Three distinct technical barriers have historically limited structural characterization of underrepresented virus systems. Size barriers, arising from depth of field limitations and Ewald sphere curvature, have prevented high-resolution analysis of giant viruses exceeding 200 nm, including plant-infecting phycodnaviruses and insect-infecting iridoviruses. Symmetry barriers have caused the loss of asymmetric information in icosahedral reconstructions, obscuring functionally important features such as portal complexes and genome packaging across all host categories. Morphology barriers have significantly impacted plant viruses, where the predominance of flexuous filamentous forms has rendered many economically important pathogens structurally intractable.

Each barrier has driven the development of targeted methodological solutions. Block-based local refinement overcomes size barriers by treating large particles as collections of smaller, independently refinable blocks, effectively bypassing physical limitations to achieve near-atomic resolution. cryo-ET overcomes symmetry barriers by reconstructing individual particles without symmetry imposition, revealing asymmetric features lost in conventional processing. STA overcomes morphology barriers by averaging locally repetitive elements within globally heterogeneous assemblies, enabling the structural determination of flexible filamentous and pleomorphic viruses.

Strategic application of these methodological solutions to underrepresented virus systems offers a clear path toward a more balanced virosphere. Block-based reconstruction can now be systematically applied to giant plant and insect viruses, while subtomogram averaging should be prioritized for the flexuous filamentous plant viruses that cause the majority of agricultural disease. While implementing these strategies requires coordinated efforts linking virology expertise with cryo-EM facilities, the ultimate goal transcends administrative metrics. Bridging this structural gap is not merely an archival necessity but a fundamental step toward deciphering the universal principles of viral assembly and evolution. By illuminating the vast unexplored diversity of the virosphere from giant insect viruses to flexible plant pathogens we secure the knowledge base required to anticipate and mitigate emerging threats across biological kingdoms.

## Figures and Tables

**Table 1:** Structural features and associated technical challenges in underrepresented viral lineages.

Target Category	Representative Models	Structural Feature	Technical Challenge	Methodological Solution
Giant Viruses	Mimivirus, Iridovirus, PBCV-1	Large diameter	Ewald sphere curvature effects	Block-Based Reconstruction (Local Refinement)
Plant Viruses	Potyviridae, Closteroviridae	Flexuous/Filamentous morphology	Conformational heterogeneity	Subtomogram Averaging (STA) or Helical Reconstruction
Insect Viruses	Caudovirales (phage-like), Podoviridae	Hidden Asymmetry (e.g., Portals)	Averaging artifacts	Cryo-ET Validation & Symmetry Relaxation

## Data Availability

No new data were created or analyzed in this study.

## List of Abbreviations

AI: Artificial Intelligence; Cryo-EM: Cryogenic Electron Microscopy; Cryo-ET: Cryo-Electron Tomography; Cryo-FIB: Cryo-Focused Ion Beam; CTF: Contrast Transfer Function; EMDB: Electron Microscopy Data Bank; HA: Hemagglutinin; HCV: Hepatitis C Virus; HIV: Human Immunodeficiency Virus; NA: Neuraminidase; NCLDV: Nucleocytoplasmic Large DNA Virus; SGIV: Singapore Grouper Iridovirus; SPA: Single-Particle Analysis; STA: Subtomogram Averaging; TMV: Tobacco Mosaic Virus.
